# Oculomotor screening and neuro-visual rehabilitation following pediatric brain tumor resection

**DOI:** 10.3233/PRM-220127

**Published:** 2024-06-26

**Authors:** Per Ertzgaard, Per Nyman, Maria Jakobsson, Jan Johansson

**Affiliations:** a Department of Rehabilitation Medicine and Department of Health, Medicine and Caring Sciences, Linkoping University, Linköping, Sweden; b Department of Pediatrics, and Department of Health, Medicine and Caring Sciences, Linköping University, Linköping, Sweden; c Department of Clinical Neuroscience, Division of Eye and Vision, Karolinska Institute, Stockholm, Sweden

**Keywords:** Pediatrics, brain tumor, vision disorders, orthoptics, oculomotor rehabilitation

## Abstract

Visual difficulties are common after brain tumors, despite a lack of visual complaints at diagnosis. These include difficulties with eye movements, visual coordination, vergence, accommodation, and photophobia, in addition to more obvious problems such as visual field defects.

This case report presents the results of a thorough neuro-visual evaluation in a boy with sequelae after a brain tumor including intermittent double vision that was not explained by routine visual examination. Subjective complaints included poor reading perseverance, intermittent blurred and double vision, headache around the eyes when performing near activities, less efficient eye movement behavior in reading tasks, and increased sensitivity to visual motion. The patient participated in a multidisciplinary visual rehabilitation program that included reading glasses with prism compensation and tinted glasses, as well as training with the aim of improving eye teaming, near vision functions, and perseverance in eye movements.

The patient responded quickly to the vision therapy program, with positive changes after just four weeks. Repeated neuro-visual evaluations over eight months showed remarkable improvements that were stable over time. This encouraging case report supports the notion that neuro-visual evaluation and rehabilitation should be included in the follow-up of patients after brain tumors.

## 1 Introduction

Brain tumors are the most common tumors in children, and they are an important cause of mortality and disability [1]. With improved treatment protocols, mortality has decreased significantly over the past few decades.

Disability in survivors includes cognitive [2], behavioral, sensorimotor, auditory, and visual problems [1, 3]. In addition, having a brain tumor has a major psychosocial impact affecting and involving the whole family, as well as activity and participation in school and leisure activities.

In recent years, awareness of visual disorders beyond the obvious, i.e., anopsia, nystagmus and strabismus, has increased. This relates to symptoms that are common in a diverse array of brain disorders such as traumatic brain injuries [4, 5], encephalitis, stroke [6] and sequelae after SARS-CoV-2 infection (post-COVID) [7]. They include difficulties with eye movements, eye teaming, vergence, accommodation, and photophobia, in addition to more obvious problems such as visual field defects [4]. These difficulties may impair school performance severely [8–10].

In children with primary brain tumors, it was found that 90% had abnormal findings in a comprehensive ophthalmological evaluation, although only 15% had visual complaints at tumor diagnosis [11].

From a rehabilitation perspective, it is often possible to compensate for or train these visual dysfunctions with improvements in reading speed and endurance [12–14], thereby facilitating better school performance [15]. Visual impairment can also affect balance and postural control [16], thereby interfering with stability in gait and standing.

The pediatric Brain Tumor Team at H.R.H. Crown Princess Victoria Children’s and Adolescents’ Hospital in Linköping is part of one of six specialized pediatric oncology centers in Sweden. The clinic has a multidisciplinary standardized follow-up at three months, two years, and five years after surgery, with a final visit at the age of 18.

In accordance with encouraging results in treating visual disorders in diffuse brain disorders, the clinic has now included children that have been affected by brain tumors in a neuro-visual rehabilitation program (Table 1). This case report presents the first child participating in this program.

**Table 1:** Structure of the neuro-visual rehabilitation program

	Activities	Responsible party
Screening	History of visual problems	MD (pediatric rehabilitation)
	Convergence Insufficiency Symptom Survey (CISS)	Nurse, occupational therapist, or special education teacher with training in visual screening MD (pediatric rehabilitation)
	Vestibular-Ocular Motor Screening (VOMS)
	Convergence, accommodation, visual motion sensitivity, saccades, pursuit eye movements
Neuro-visual examination	Visual acuity at near and far, eye motility, eye movement assessment, stereovision, binocular vision function, accommodation, visual motion sensitivity	Optometrist with experience in neuro-visual evaluation
Vision therapy	Individualized treatment including problem-directed training activities, and compensatory measures such as glasses with or without prism and tinted lenses	Optometrist
	Regular follow-up and prompting of training activities	Occupational therapist and nurse with training in visual rehabilitation
	Neuro-visual examination
	Adjustment of vision therapy program
Evaluation	Neuro-visual examination	The whole team
	Adjustment of vision therapy program

## 2 Case report

In 2014, a previously essentially healthy nine-year-old boy came to the pediatric clinic due to a few months’ symptoms of headache, dizziness, balance disorders, and coordination difficulties in the right arm. There were no subjective visual complaints.

**Fig. 1:**
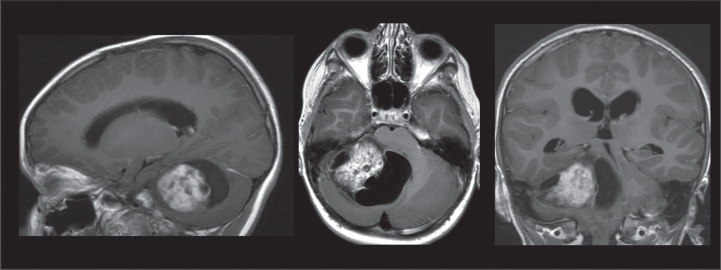
MRI findings of a tumor in the right cerebellar hemisphere. T1 weighted sequences in a sagittal, axial, and frontal view.

An MRI showed a tumor in the right cerebellar hemisphere, 5.5×4.5×4.5 cm, with a solid component of 2.5×3×3.5 cm. The tumor caused compression of the fourth ventricle with hydrocephalus, deformed the pons and mesencephalon, and herniated both the right part of the ambient cistern via the tentorial opening and the right cerebellar tonsil in the foramen magnum (Fig. 1).

Neurosurgical extirpation of the cerebellar tumor was performed with macroscopic radicality. A subsequent MRI confirmed that there was no tumor remnant. Postoperative recovery was complicated by cerebrospinal fluid (CSF) leakage occurring 10 days post-surgery. Revision and closure of the CSF leakage and lumbar drainage for five days was followed by mobilization without further complications.

Histopathological examination of the tumor showed a pilocytic astrocytoma. This is the most common type of brain tumor in children, with a low World Health Organization (WHO) grade of I [17], which indicates the resemblance of normal cells and slow growth. Radiological control revealed no residual tumor; therefore, no further oncological treatment was indicated.

Evaluations, including MRIs, were done every six months during the first year and then once a year until at least five years after surgery, with no sign of tumor recurrence.

### 2.1 General assessment

At follow-up, the patient’s parents described him as active and not particularly limited in everyday life. This included being able to resume training in American football and soccer less than one year postoperatively. Despite improvements in motor function, there was still a right-sided impairment in which the right hand responded more slowly and fine manipulation was more difficult, as well as discrete difficulties in both static and dynamic balance. When visiting the team five years after surgery, it also emerged that he had problems cycling because he felt insecure and scared and was unable to start by himself. Before the tumor, he cycled without difficulty. After specific training with a physiotherapist, the boy learned to ride a bike again without much effort, indicating motivation to train to improve function.

Neuropsychological assessments were performed every two years starting one year post-surgery. The results of the assessments are presented in Table 2. To summarize, the results were slightly under average with some improvements over the years. In every assessment, he had difficulties with complex figure tasks, especially regarding three-dimensional figures and perspective displacements. The approach he used when copying a complex image indicated that he had difficulty obtaining an overview and creating a strategy for the task.

**Table 2:** Results of neuropsychological testing using selected items from the Wechsler Intelligence Scale for Children (WISC). Numbers are presented as scaled scores with a population average of 10, except for full-scale IQ in which the average is 100. In 2015, the WISC-IV was used, while the WISC-V was used for the remaining years, limiting the validity of comparisons between the years

	2015	2017	2019	2021
Similarities	12	12	nt	10
Vocabulary	10	8	8	8
Block design	9	11	nt	nt
Matrix reasoning	7	7	9	10
Digit span	6	8	8	8
Coding	5	7	8	8
Symbol search	12	nt	9	9

nt = not tested.

He reported having difficulty finding words at times. He also pointed out that he got tired when there was a lot of talk at school and described himself as sensitive to sound.

At medical check-ups, double vision was noted. An ophthalmologist was consulted and, at repeat visits (2018 and 2019), he continued to report both monocular and binocular double vision, although neither the ophthalmologist nor an orthoptist could find the cause of this, as eye examinations and vision tests were normal. The boy experienced some improvement (less double vision) with corrective lenses and eyeglasses were recommended. During the last years of follow-up, there were no further visits to the eye clinic, but intermittent problems with double vision were still reported.

## 3 Neuro-visual evaluation

The patient’s main symptoms were poor reading perseverance, intermittent blurred and double vision, and headache around the eyes when performing near activities. He scored 41 on the Convergence Insufficiency Symptom Survey (CISS, 0–60, normal = below 16) [18]. He also reported discomfort from ambient light, in particular the lighting in the classroom, along with increased sensitivity to visual motion as in busy environments or when traveling as a passenger in a car.

A vision examination was performed by an optometrist at the clinic which demonstrated normal uncorrected visual acuity, i.e., no need for eyeglasses to see clearly at distance. An assessment of near vision found a combined convergence and accommodative insufficiency, meaning that the ability to maintain clear single (not double) vision, e.g., when reading, was impaired. The Developmental Eye Movement Test (DEM) [19] showed increased duration and ratio, indicating less efficient eye movement behavior in the simulated reading task. Furthermore, increased sensitivity to visual motion was confirmed with the VMS (Vision Motion Sensitivity) Clinical Test Protocol [20]. For details, see Table 3 (vision exam 1).

**Table 3:** Findings in vision examinations. For further details on eye teaming and accommodation, see Figs. 2–5

	2021-10-13	2021-12-15	2022-03-02	2022-06-08
Visual function	Initial examination	2 month follow up	4 month follow up	8 month follow up
Visual acuity, decimal
Right eye	1.0		1.0	1.0
Left eye	1.0		1.0	1.0
Near visual acuity	6 p (reduced)		5 p (normal)	5 p (normal)
Stereo acuity, Lang II test	Pos		Pos	Pos
Eye motility	NAD		NAD	NAD
Eye movements
Pursuit eye movements	NAD		NAD	NAD
Pro-saccades	Slowed latency		NAD	NAD
Self-paced saccades	Moderate pace, discomfort		NAD	NAD
Eye teaming
Cover test, far	Ortho		Ortho	Ortho
Cover test, near	Ortho		Ortho	Esophoria 1 base out (normal)
Developmental eye movement test
Test A+B (seconds)	35		32	30
Test C (seconds)	51		37	33
Ratio	1.4 (increased ratio)		1.1 (normal ratio)	1.1 (normal)
VMS clinical test protocol
Right visual field	6.5	6.5	6	7
Left visual field	4	5	7	6
Lower visual field	8	8	8	5
Upper visual field	4	7	6	3
Total score	22.5	26.5	27	21

NAD: no abnormality detected.

### 3.1 Intervention

As a first step, reading glasses were prescribed to ease the effort of performing near vision tasks, thereby alleviating symptoms when doing schoolwork. The reading glasses were dispensed with a dioptric power of +1.00 and a 1.5 base in prism bilaterally to provide oculomotor and accommodative relief. In addition, the reading glasses were provided with a tint, FL41 medium, based on clinical try-out. The patient was encouraged to use the glasses in all near vision tasks but not during the vision therapy program, which was initiated with the aim of improving eye teaming, near vision functions, and perseverance in eye movements.

All exercises were initially performed seated and later on while standing, if they could be done safely with respect to postural stability. Eye teaming and near vision functions were targeted with therapies including Brock’s string, pen push-up, Hart chart facility task, and concentric circles. Therapies targeting eye movement included pursuit eye movements, saccades, and head turns while maintaining fixation. The training activities were performed daily at home by the patient after receiving instructions from a nurse and an occupational therapist with experience in the field, who also did follow-up evaluations. The exercises and their implementation during the four-month program are described in detail in the supplement.

### 3.2 Outcome

**Fig. 2:**
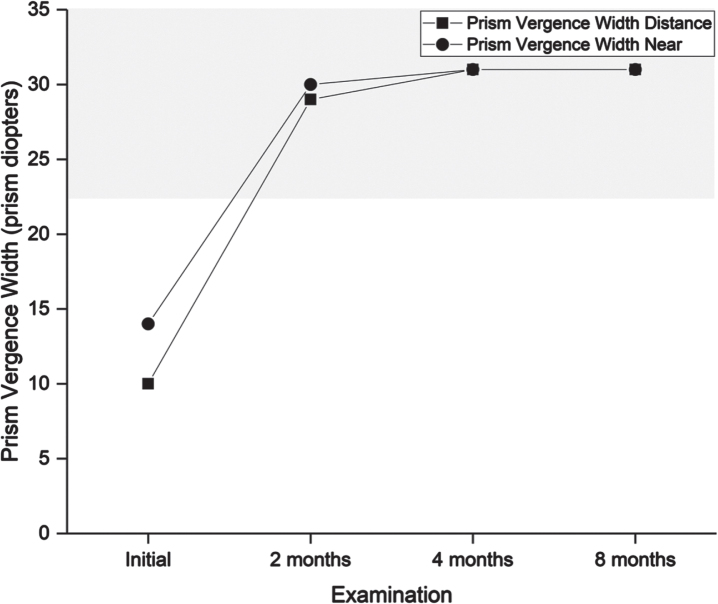
Prism vergence width measured in prism diopters. A higher value is better. The grey shaded area indicates the normal range. Prism vergence width is relevant for estimating the ability to compensate any heterophoria (latent squint) and perseverance in binocular coordination. A suboptimal prism vergence may lead to unstable vision, asthenopic symptoms, and intermittent double vision.

**Fig. 3:**
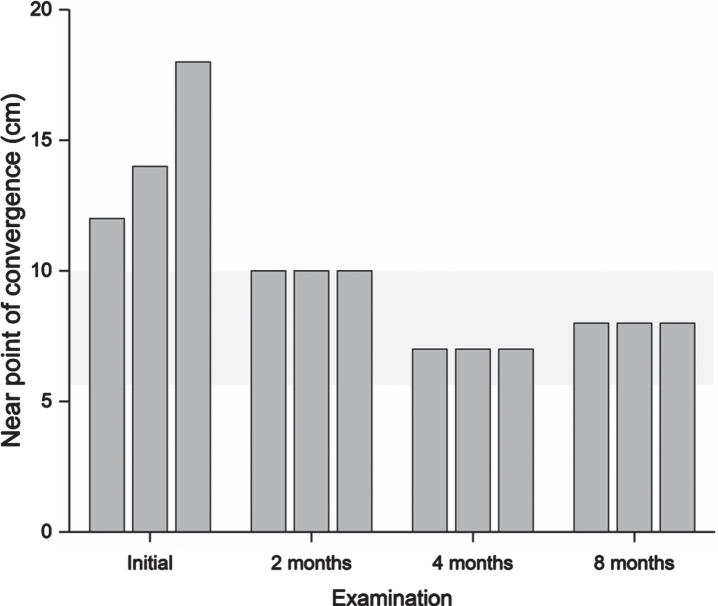
Near point of convergence measured in centimeters. A lower value is better. The grey shaded area indicates the normal range. The measurement was repeated three times to detect signs of fatigue. At examination one, clear signs of fatigue can be seen, where the value increases for each subsequent measurement. Near point of convergence is relevant for estimating the ability to focus the eyes for near activities. A suboptimal near point of convergence may lead to asthenopic symptoms and intermittent double vision when performing near activities, e.g., when reading.

**Fig. 4:**
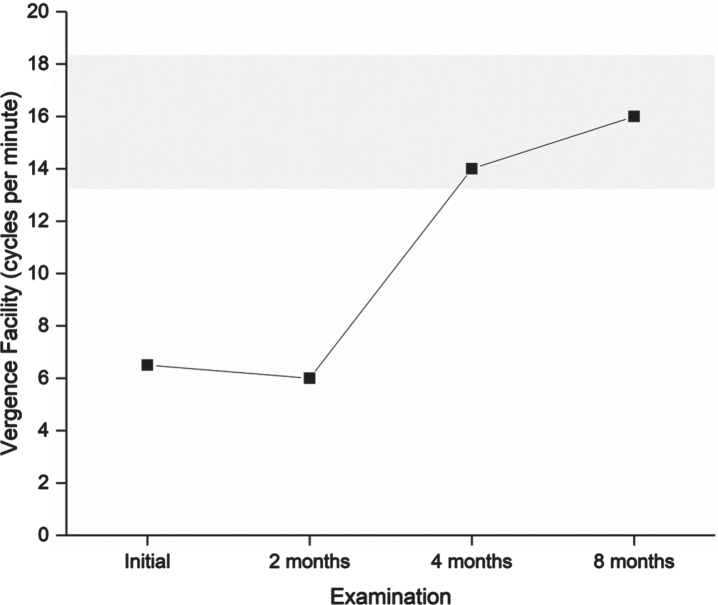
Vergence facility measured in cycles per minute. A higher value is better. The grey shaded area indicates the normal range. Vergence facility is relevant for estimating the ability and perseverance to effortlessly alter focus between near and distance. Suboptimal vergence facility may lead to sluggish refocus and asthenopic symptoms including headache when performing activities that require continuous refocus, e.g., in traffic situations, meetings and schoolwork.

**Fig. 5:**
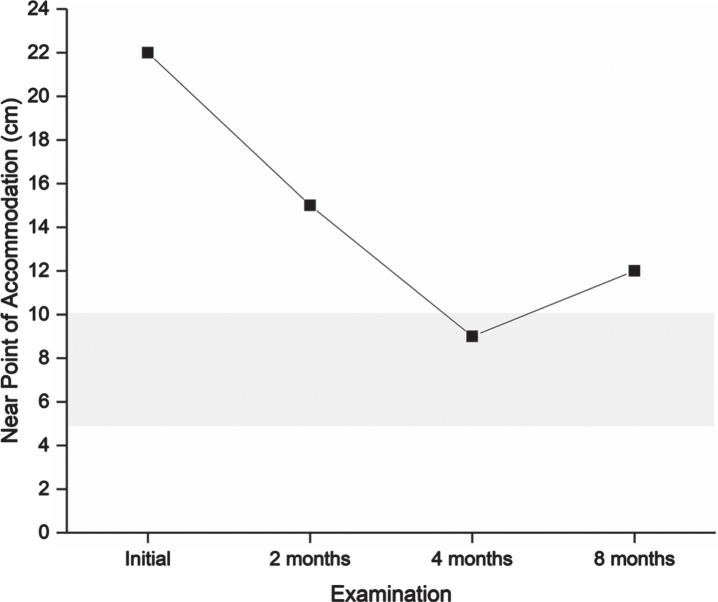
Near point of accommodation measured in centimeters. A lower value is better. The grey shaded area indicates normal range according to age. Near point of accommodation is relevant for estimating the ability and perseverance to maintain focus on near activities. A suboptimal near point of accommodation may lead to blurred vision and asthenopic symptoms close up, e.g., when reading.

Follow-up examinations took place two, four, and eight months after initiating the program (Table 3, Figs. 2–5).

After completing the four-month program, the patient scored 35 on the CISS. Symptoms were reduced by at least one step for nine items: tired eyes, uncomfortable eyes, headache, difficulty remembering when reading, sense of floating words, slow reading, sore eyes, in and out of focus, and the need for re-reads. He described less frequent headaches, less blurred vision both near and far, and, to some degree, less discomfort from movement in the surrounding environment. The final vision examination at the end of the program found normalized performance in all targeted visual functions (Figs. 2–5), and he was instructed to halve the intensity of the therapy for another four weeks and then finish. The DEM showed normalized reading duration and ratio, indicating that simulated reading eye movements were performed with greater efficiency. The VMS Clinical Test Protocol still indicated increased sensitivity to visual motion. The patient was therefore encouraged to continue daily activities in which he would be exposed to motion in the environment. The reading glasses were no longer necessary, but he was advised to keep them for use when needed. At the last follow-up examination three months after ending the step-down aspect of the vision therapy program, all visual function measures were maintained apart from accommodation, in which a slight reduction in maximum accommodative amplitude was found. The CISS continued to improve to 29.

## 4 Discussion

The patient responded quickly to the vision therapy program and improvements were observed after just four weeks of training. Reading glasses were dispensed at an early stage, which facilitated schoolwork and vision therapy by reducing visual discomfort. The patient described significant relief with tinted lenses, especially when doing reading and writing tasks in the classroom. These improvements helped motivate the patient and thereby increased compliance to the neuro-visual rehabilitation program.

Accommodation improved during the vision therapy program, with a slight reduction at follow-up which may have been temporary due to workload during the day. The patient reported using the glasses less frequently and had also finished exercises targeting accommodation. Other visual function measures were maintained, indicating that accommodation may require longer or ongoing intervention. It should also be remembered that it is necessary to balance interventions against other daily demands such as schoolwork. In this case, glasses for distance viewing with a subtle change in power towards the lower part of the glass were recommended. The intention is to relieve strain of near work. These glasses are sometimes called anti-fatigue and adaptation to them is generally easy.

Visual symptoms with near activities, estimated with the CISS, were reduced from a score of 41 to 29. For children up to 18 years of age, a score of 16 is considered to indicate a normal level of symptoms [18]. The items that were still graded as ‘occurring frequently’ or ‘always’ were losing concentration while reading, difficulty remembering, slow reading, losing one’s place while reading, and re-readings. Given the complexity of the reading process, functions other than purely visual ones may have influenced the outcome. The findings highlight the need for multi-professional assessment and intervention.

The patient still obtained high scores on the VMS Clinical Test Protocol, indicating that an increased sensitivity to movement in the environment remained. This phenomenon can cause discomfort and have a fatiguing effect, and treatment options include further vision therapies such as desensitization. In parallel, it is important to consider compensatory measures to provide relief in daily activities. These include wearing cover frames while travelling or residing in busy environments, having access to a quiet space to focus on schoolwork, and bi-nasal occlusion in severe cases.

Visual sequelae after treatment for brain tumors are underestimated [11]. This case report indicates that neuro-visual examination and rehabilitation should be considered for patients after treatment for brain tumors similar to those with other brain disorders [4–6]. The strength of this case report is that the patient was in a stable situation in which no spontaneous improvement could be expected and any improvement would most likely depend on the intervention. In this case study, it is not possible to elaborate on which components (i.e., one of the six tasks, the combination of all six tasks, or tasks plus reading glasses with tint) were responsible or essential for the observed gains. Future research would need to include a component analysis to make such conclusions. In addition, a systematic prospective study is necessary to understand for whom the effects of a neuro-visual rehabilitation program are most advantageous.

## 5 Conclusion

A patient who may not have severe enough difficulties to score below average in routine visual testing may still need considerable neuro-visual support. This case report highlights that oculomotor and accommodation disorders can exist despite normal vision and ophthalmological examinations. Seemingly well-functioning patients may have major concerns, especially in activities with high demands on vision, like participation in school. This case supports the necessity of a multidisciplinary approach to capture the scope of the problem and establish a strategy for training and adaptations that can have a mitigating effect. For this reason, oculomotor screening in multidisciplinary follow-up is encouraged to detect individuals in need of specific eye training.

## Supplementary Material

Supplementary Material
