# Supplementary material for: Oculomotor screening and neuro-visual rehabilitation following pediatric brain tumor resection
Source: J Pediatr Rehabil Med. 2024 Jun 26;17(2):253–60. doi: 10.3233/PRM-220127 (PMC11307090; doi:10.3233/PRM-220127)
Supplement: Supplementary Material [file prm-17-prm220127-s001.docx]

# Supplement

**Description of visual exercises and their implementation**

In the Brocks string exercise, a 1-meter string with 3 beads with different colours was used. The exercise was introduced by demonstrating the concept with only one bead to ensure that the patient could perceive the crossed lines passing through the bead due to physiologic diplopia. In the initial phase of the exercise, the position of the nearest bead was decided by finding the nearest point at which the patient could maintain single vision, see the bead single not double. The second and third beads were then positioned approximately 15-20 cm apart beyond the most proximal bead. The patient was encouraged to maintain correct focus on each bead before switching focus to the next. The goal was to maintain correct focus on each bead for 10 seconds and with training bring the most proximal bead to 10 cm from the eyes. The exercise was performed for 90 seconds and repeated three times with the patient being encouraged to perform as many correct refixations as possible (max 10 seconds each) during each 90 second session.

In the Pencil push-up exercise the patient held the pen approximately 40 cm from the eyes as a starting point. He was then encouraged to bring it as close as possible (push-up), at a steady pace, while maintaining single vision, that is, seeing the pen as single. Once the pen was seen double the instruction was to slowly back out again while trying to regain single vision. During the introduction to the exercise the therapist observed the eyes of the patient and provided feedback to maintain fixation on the pen if necessary. The goal was to bring the pen to 6–10 cm from the eyes, always seeing it single. The exercise was performed for 90 seconds and repeated three times and the patient was encouraged to perform as many push-ups as possible during each 90 second session.

Hart charts consists of two charts with rows of letters, one small and one large were the smaller one is held in the hand and the larger one can be seen from 2-3 meters. It is used to train the flexibility of eye teaming and focusing at near. The instruction was to read one line of letters on the near chart, then swiftly alter focus to the distance chart and read the corresponding line of letters, then back to the near chart and read the next line, until all lines had been read. It was emphasized that the patient should achieve clear vision as soon as possible after switching focus and not switch back until clear vision was achieved. The exercise was repeated for three times with the final goal to complete it in approximately one minute.

Eccentric circles were used to train eye teaming at distance viewing. The circles (outer diameter 20 cm, printed on A4-sheets) were attached on the wall at eye level. The patient was positioned, approximately 3 meters from the wall looking directly at the circles. The patient was instructed to over-converge the eyes slightly, that is eyes pointing towards nose as if looking at something very close. At the same time the patient was encouraged pay attention to the perceptual effect happening to the circles on the wall, that is, to perceive a third, centred, set of circles and to see the word “clear” clearly. If the patient had difficulties to maintain the over-converged focus, the therapist demonstrated how to use his/her own thumb as fixation support. In the beginning the patient was encouraged to try to achieve and maintain focus as many times as possible during one minute at a time. The final goal was to maintain correct focus for 10 seconds for 10 repetitions and to be able to repat this for three sessions.

Eye movement perseverance was targeted with free-space saccadic and pursuit exercises. The saccadic exercises were performed with distinct visual targets on a wall at 2-3-meter distance. For visual targets post-it notes with letters were used. They were placed in a square with the upper pair at eye-level and the lower one meter below. The distance from right to left were 1.5 meter. In the first phase the exercise was performed at own pace with accuracy as the priority. In the next phase the pace was increased stepwise, and a metronome used as support to keep the pace. The exercise was performed for 1 minute with three repetitions.

In the pursuit exercises the patient held a stick with a distinct visual target and moved it in a pattern back and forth at eye level. It was emphasized that the eyes should always follow and maintain focus on the target. It was first done at self-chosen pace and further on slightly faster and wider movement was encouraged. To increase the challenge the patient was encouraged to perform the exercise with a background with more irregular patterns or clutter. The exercise was performed for 1 minute with three repetitions. For both saccadic and pursuit exercises it was emphasized that the patient’s head should be stationary while moving the eyes.

The exercise targeting visual fixation during head movement was done by having the patient fixating a distinct visual target while making head turns sideways, as if shaking the head ‘no’. Initially, it was emphasized to perform the head movements at own pace while carefully maintaining clear focus on the visual target. The patient was then encouraged to increase pace. The exercise was performed for 30 seconds and repeated three times.

All exercises were performed seated initially and further on in standing position, provided that it could be done safely with respect to postural stability.
